# Comparing serum ferritin levels during COVID-19 infection and recovery period in pediatric patients with transfusion-dependent thalassemia, a single-center study

**DOI:** 10.3389/fmed.2023.1056599

**Published:** 2023-02-09

**Authors:** Wulandewi Marhaeni, Fabiola Vania Felicia, Arvin Leonard Sumadi Jap, Edi Hartoyo, Pudji Andayani

**Affiliations:** ^1^Division of Hematology and Oncology, Department of Pediatrics of Ulin General Hospital, Faculty of Medicine, Universitas Lambung Mangkurat, Banjarmasin, Indonesia; ^2^Department of Pediatrics of Ulin General Hospital, Faculty of Medicine, Universitas Lambung Mangkurat, Banjarmasin, Indonesia; ^3^Division of Tropic Infection, Department of Pediatrics of Ulin General Hospital, Faculty of Medicine, Universitas Lambung Mangkurat, Banjarmasin, Indonesia

**Keywords:** transfusion-dependent thalassemia, thalassemia, pediatric, hyperferritinemia, ferritin, COVID-19

## Abstract

**Background:**

Ferritin has been recognized as a predictor of severity among Coronavirus-19 disease (COVID-19) patients. Studies have shown higher levels of ferritin in patients with COVID-19 than in healthy children. Patients with transfusion-dependent thalassemia (TDT) basically have high ferritin level due to iron overload. It is uncertain whether serum ferritin level in these patients is associated with COVID-19 infection.

**Objective:**

To evaluate ferritin levels in TDT with COVID-19 before, during, and after the course of infection.

**Methods:**

This retrospective study enrolled all TDT children with COVID-19 infection that were hospitalized in Ulin General Hospital Banjarmasin during the COVID-19 pandemic (March 2020 to June 2022). Data were collected from medical records.

**Results:**

There were 14 patients included in this study, 5 patients had mild symptoms and 9 patients were asymptomatic. The mean of hemoglobin level upon admission was 8.1 ± 3 g/dL and serum ferritin level were 5148.5 ± 2651.8 ng/mL. The average serum ferritin level during COVID-19 infection was 2373.2 ng/mL higher than before infection and then decreased by 952.4 ng/mL after infection. We found no association of increasing serum ferritin with patients’ symptoms (*p* = 0.27). The severity of anemia also was not correlated with the presentation of COVID-19 infection (*p* = 0.902).

**Conclusion:**

Serum ferritin levels in TDT children may not reflect disease severity or predict poor outcomes during COVID-19 infection. However, the presence of other co-morbid conditions/confounders warrants cautious interpretation.

## 1. Introduction

Coronavirus disease 2019 (COVID-19) pandemic has been going on since December 2019 and affected many people from neonates to elders (1, 2). Clinical symptoms of COVID-19 vary from asymptomatic infection to severe form of the acute respiratory syndrome, even death (3). Although SARS-CoV-2 infection in children was less severe as compared to adults, children with comorbid disease may develop severe disease (4, 5). Hematological disease has been known as one of the risk factors related to the severity of COVID-19 (5, 6).

β-thalassemia is one of the most common hematological diseases in the world, with approximately 1.5% of the global population carrying the β-thalassemia trait (7, 8). β-thalassemia is divided into two groups: transfusion-dependent β-thalassemia (TDT) and non-transfusion-dependent β-thalassemia (NTDT) (6, 9). TDT children might be at risk for severe disease of COVID-19 because of several complications like iron overload, ineffective erythropoiesis, hypercoagulability, splenectomy, and multiple organ damage due to excess iron (6, 10). These complications may increase the susceptibility to infection in TDT children (11). Ferritin is a marker for iron storage status in humans. High serum ferritin levels indicate increased or normal iron storage (12). Ferritin also known as an acute phase reactant, is elevated in acute and chronic inflammation and/or infection (12, 13). In cases of COVID-19, ferritin has been correlated with disease severity that may serve as a biomarker (14, 15). Measurement of serum ferritin is used to diagnose iron overload in β-thalassemia children (16). Thus, ferritin levels in these children are already elevated due to iron overload (9).

No study compares serum ferritin levels during COVID-19 infection and recovery period in pediatric patients with TDT. In this study, we aim to compare and analyze serum ferritin levels in children with TDT and COVID-19. Clinical severity and hemoglobin levels were also described.

## 2. Materials and methods

### 2.1. Methods and study criteria

A single-center retrospective study was conducted in Ulin General Hospital, Banjarmasin. We included all TDT children aged 0–18 years that were diagnosed with confirmed COVID-19 at Ulin General Hospital Banjarmasin between June 2020 and June 2022. Data were collected from the isolation registry book and patients’ medical records. Incomplete medical record data were excluded from the study.

### 2.2. Operational definitions

TDT is defined as the need for repeated blood transfusion regularly with the assumption of 2–4 units of packed red cells monthly (17). Diagnosis COVID-19 was confirmed by SARS-CoV-2 polymerase chain reaction (PCR) test results. Clinical symptoms were considered present if developed at the beginning of hospitalization to discharge. Laboratory data collected are the laboratory results before, during, and after COVID-19. The results of laboratory data before and after COVID-19 were collected from previous and afterward hospitalization, which was 1–two 2 months before and after COVID-19 infection.

### 2.3. Outcome

The outcome of this study was serum ferritin levels before, during, and after COVID-19 infection among children with TDT. Presentation of clinical symptoms, hemoglobin levels, and association of serum ferritin levels in TDT children with COVID-19 were also of interest.

### 2.4. Statistical analysis

Baseline characteristics were reported using frequencies, percentages, mean and ranges. Independent *t*-test was used to determine the association between numeric variables. All statistical analyses were done using Statistical Package for the Social Sciences (SPSS) version 23. Results were considered statistically significant if α < 0.05. Informed consent was obtained from all parents of the study participants. This study was approved by the Ethics Committee of Ulin General Hospital (register number 198/X-RegRiset/RSUDU/22).

## 3. Results

A total of 15 children with TDT were reported to have COVID-19 infection from 205 thalassemia children registered in Ulin General Hospital. Generally, there were 65 patients diagnosed with confirmed COVID-19 infection and hospitalized at Ulin General Hospital from June 2020 to June 2022. One child was excluded because of incomplete laboratory data. The characteristics of these patients was described in Table 1.

**TABLE 1:** Baseline characteristic.

Characteristic	Total
**Gender (*n*, %)**	
Male	11 (78.6)
Female	3 (21.4)
Age (years) (mean, SD)	11 ± 5
**Symptoms (*n*, %)**	
Symptomatic	5 (35.7)
Asymptomatic	9 (64.3)
**Iron chelating agent (*n*, %)**	
Deferiprone (DFP)	8 (57.1)
Deferasirox (DFX)	6 (42.9)
**Hemoglobin (g/dL) (mean, SD)**	
Pre-COVID-19	9 ± 2.1
During COVID-19	8.1 ± 3
Post-COVID-19	8.4 ± 1.9
**White blood cell (/uL) (mean, SD)**	
Pre-COVID-19	7735.7 ± 2389.9
During COVID-19	6928.6 ± 3813.2
Post-COVID-19	8293 ± 3243.3
**Platelet (10^3^/uL) (mean, SD)**	
Pre-COVID-19	256.3 ± 143.3
During COVID-19	281.3 ± 123.1
Post-COVID-19	267.6 ± 151.2
**Ferritin (ng/mL) (mean, SD)**	
Pre-COVID-19	3934.5 ± 1804
During COVID-19	5148.5 ± 2651.8
Post-COVID-19	4264 ± 2516.1
**Ferritin changes (ng/mL) (mean, SD)**	
Pre- vs. during COVID-19	2373.2 ± 1320.9
During vs. post-COVID-19	952.4 ± 2978.1
**Radiologic findings (*n*, %)**	
Pneumonia	6 (42.9)
Normal	8 (64.3)
**Outcome (*n*, %)**	
Self-isolation	11 (78.6)
Recovered	3 (21.4)

The youngest TDT child with COVID-19 infection was 1-year old and the oldest was 18 years old. No patient had severe signs and symptoms of COVID-19 infection in this study. Five patients (35.7%) were symptomatic. Fever presented in 4 patients (80%) and other symptoms were cough (40%), anosmia (20%), ageusia (20%), and myalgia (20%).

The range of hematological parameters from 14 patients upon admission were hemoglobin 2.6 to 13.2 g/dl, white blood cell (WBC) 2400 to 18700/μL, platelet 73 to 441 × 10^3^/μL, and lymphocyte count 1210 to 3430/μL, respectively. Serum ferritin levels were 980.51 to 8974.82 ng/mL. The average serum ferritin level changes in TDT children during COVID-19 infection was 2373.2 ng/mL higher than before infection and after COVID-19 infection was 952.4 ng/mL lower than during infection. Six patients had pneumonia from radiological findings, two (33.3%) of which were asymptomatic and none needed oxygen supplementation.

The comparison of serum ferritin level changes before and during COVID-19 infection vs. during and after COVID-19 infection were statistically significant (*p* = 0.003; mean differences 3371.84 ng/mL; 95% CI 1421.26 to 5322.43). Although there were increasing serum ferritin levels in TDT children during COVID-19 infection, these changes were not associated with the patient’s symptoms (*p* = 0.27), and no severe disease was presented. The severity of anemia also was not correlated with the presentation of COVID-19 infection (*p* = 0.902). Data were presented in Table 2. We also found that the serum ferritin elevation was not associated with pneumonia (*p* = 0.554).

**TABLE 2:** Laboratory changes in thalassemia children with confirmed COVID-19.

Laboratory variables	Symptomatic (*n* = 5)	Asymptomatic (*n* = 9)	*P*-value	95% CI
Hemoglobin (g/dl)	8.22 ± 1.48	8.04 ± 3.68	0.902	−2.88 to 3.23
Ferritin (mg/dl)	6235.1 ± 3075	4544.8 ± 2356.2	0.27	−1491.14 to 4871.65

## 4. Discussion

Serum ferritin levels in transfusion-dependent β-thalassemia patients were higher compared to healthy people as a result of regular transfusions (9, 18, 19). The higher level of ferritin was directly associated with iron accumulation in tissue which commonly involves the heart, liver, lungs, and endocrine glands (12, 18, 20). During iron overload, transferrin saturation percentage that leaves non-transferrin-bound iron as free iron. This free iron enters some cells and pooled within the cells. Intracellular iron is stored either in ferritin or in hemosiderin (12, 13). Ferritin plays a major role in iron homeostasis, which acts as the main iron storage mechanism and releases iron when the level decreased (12). Therefore, ferritin is used to identify iron overload in β-thalassemia patients and the need for iron chelation agent therapy initiation. Iron chelation agent was indicated if serum ferritin levels exceed 1000 ng/mL in TDT children (17, 21). The raise of serum ferritin above 1000 ng/mL is considered hyperferritinemia (21, 22).

Ferritin also represents an acute-phase protein that is elevated in the course of inflammation, both infectious and non-infectious (14, 21). In inflammatory conditions, serum ferritin may be within normal or increased even though the actual iron stores are absent. Increased ferritin levels are associated as a biomarker of cell damage, oxidative stress, the presence of disease processes, and the severity of chronic disease processes (23). Hyperferritinemia in inflammatory and infective disorders is believed to be cytokine-mediated that implicate interleukin (IL) 1β, IL-6, IL-18, interferon (IFN)-γ, tumor necrosis factor-α (TNF-α) and macrophage-colony stimulating factor (22). These pro-inflammatory cytokines induced the liver to produce several proteins including C-reactive protein (CRP) and ferritin. Ferritin sources during inflammation include macrophage secretion and damaged cells. Also, ferritin may have an active pathogenic role by stimulating pro-inflammatory pathways (24). It means that there might be a feedback mechanism between cytokines and ferritin (25).

While serum ferritin in normal children represents a biomarker of cell damage, TDT children already present with high serum ferritin levels. A high level of ferritin serum was identified in those patients with COVID-19 with severe disease and poor outcomes (23, 24). In this study, an increase in serum ferritin in TDT children was observed. However, this noticeable increase before and during COVID-19 infection, although was statistically significant, was not correlated either with disease severity or poor outcome, statistically. A nationwide study in Iranian adults showed no difference of serum ferritin level between the death group and the recovery group of thalassemia patients with confirmed COVID-19 (20).

SARS-CoV-2 virus primarily affects the respiratory system with wide range of symptoms, from asymptomatic or mild nasopharyngeal symptoms to severe pneumonia. Hemoglobin disorders, particularly thalassemia, are not directly related with respiratory conditions. However, thalassemia patients are divided into three risk groups of developing severe SARS-CoV-2 infection. Highest risk group included thalassemia patients with 2 or more of the following criteria: age more than 50 years, suboptimal transfusion pre-transfusion Hb < 7.0 g/dL (currently and in the last 2–3 years), serum ferritin >4000 ng/mL (currently and in the last 2–3 years), one or more comorbidity such as diabetes, cardiac, endocrine, respiratory or hepatic disease, splenectomy with at least one comorbidity (26). Several studies showed that the susceptibility to severe disease and mortality were more profound in non-TDT patients (7, 8, 20). Thus, patients with TDT were associated with less severe disease and improved outcomes (27). These patients regularly received blood transfusions which could decrease the occurrence of anemia-related complications and compensation for bone marrow expansion. Iron chelation therapy also reduces iron overload related to multiple transfusions (27). Severe diseases of COVID-19 in TDT patients are shown to be due to the coexistence of organ damage associated with iron overload including cardiomyopathy and heart failure (5, 28).

Several possible explanations were suggested for the less severe disease and susceptibility to COVID-19 in thalassemia patients. During SAR-CoV-2 infection, the beta chain of Hb was attacked, resulting separation of iron from the porphyrin ring, and impairing the oxygen transfer process. It could be that the viral protein competes with iron to bind to this porphyrin ring, interfering the normal heme anabolic pathway (27, 29). A study had suggested that some proteins of this coronavirus attacked the heme on the beta chain of hemoglobin, both oxygenated and deoxygenated hemoglobin. The higher the hemoglobin content, the higher risk of disease. However, in case of abnormal hemoglobin (abnormal structure of Hb), it is not clear whether the viral proteins could attack this heme and then bind with this porphyrin to form a complex (29). Further research is needed to verified this theory. A national survey in French reported 16 cases of β-thalassemia patients that included 4 pediatric patients. All of pediatric patients were having few signs and symptoms during the disease course. Furthermore, the severity of COVID-19 in β-thalassemia patients, young and middle-aged patients, remained mild to moderate (30). A meta-analysis determined that the incidence rate of TDT patients with COVID-19 infection, in general, was 1.28/100,000 people per day. This number was surprisingly lower than the incidence rate in the general population which was 2.89/100,000 people per day (6).

Other hematological and biochemical parameters were also associated with poor outcomes in COVID-19 patients. A meta-analysis showed that lymphopenia (lymphocyte count < 1500/μL; OR 3.33; 95% CI: 2.51–4.41), thrombocytopenia (platelet level < 150 × 10^3^/μL; OR 2.36; 95% CI: 1.64–3.40), elevated AST (>40 IU/L; (OR 2.36; 95% CI: 1.64–3.40) and elevated AST (>40 IU/L; OR 1.71; 95% CI: 1.32–2.20), elevated creatinine (>1.18–1.5 mg/dL; OR 2.84; 95% CI: 1.8–4.46) were associated with poorer outcome (31). In this study, we have three patients with thrombocytopenia, two patients with lymphopenia, five patients with elevated AST and ALT, and one patient with elevated creatinine. However, none was presented either with severe disease nor poor outcome. Previous study reported that significant mortality rate was observed when the platelet count dropped below 100 × 10^3^/μL. Mortality rates were 61.2% with platelet counts 50–100 × 10^3^/μL and 92.1% with platelet counts < 50 × 10^3^/μL (32). Another study about lymphopenia and COVID-19 severity was observed below 800–1100/μL (33). As for the unavailable results of AST, ALT and creatinine in this study, were not provided because of the incomplete data.

This study has several limitations such as the small number of participants because the incidence rate of COVID-19 infection in β-thalassemia was shown to be lower than in the general population. As for disease severity, children were generally present with mild disease and many of thalassemia patients didn’t visit the hematology-oncology clinic in our center during COVID-19 pandemic. This mild presentation also happens in TDT children, as seen in our study. Therefore, we couldn’t observe how far the “hyperferritinemia” state occurs in TDT children with severe disease.

## 5. Conclusion

Hyperferritinemia is usually observed in TDT children due to iron overload. Our data suggest that ferritin in TDT children may not reflect disease severity or predict poor outcomes during COVID-19 infection. However, the presence of other co-morbid conditions/confounders warrants cautious interpretation. Further studies with more participants are required to determine if there’s any role of ferritin for the assessment of disease severity and outcome.

## Data availability statement

The raw data supporting the conclusions of this article will be made available by the authors, without undue reservation.

## Ethics statement

The studies involving human participants were reviewed and approved by the Ethical Committee of Ulin General Hospital. Written informed consent for participation was not provided by the participants or their legal guardians/next of kin because: Data were collected from the isolation registry book and patients’ medical records. Written informed consent was not obtained from the minor(s)’ legal guardian/next of kin for the publication of any potentially identifiable images or data included in this article.

## Author contributions

WM: principal investigator, supervision, data curation, and writing – review and editing. FF: investigation, data curation, writing draft, software, and formal analysis. AS: investigation, data curation, writing draft, and formal analysis. EH: investigation, methodology, formal analysis, and data curation. PA: investigation and data curation. All authors contributed to the article and approved the submitted version.
